# Active Surveillance of the Trachea or Throat for MRSA Is More Sensitive than Nasal Surveillance and a Better Predictor of MRSA Infections among Patients in Intensive Care

**DOI:** 10.1371/journal.pone.0099192

**Published:** 2014-06-09

**Authors:** Hee-Chang Jang, Ok-Ja Choi, Gwang-Sook Kim, Mi-Ok Jang, Seung-Ji Kang, Sook-In Jung, Jong-Hee Shin, Byeong Jo Chun, Kyung-Hwa Park

**Affiliations:** 1 Department of Infectious Diseases, Chonnam National University Medical School, Gwang-ju, Republic of Korea; 2 Office for Infection Control, Chonnam National University Hospital, Gwang-ju, Republic of Korea; 3 Department of Laboratory Medicine, Chonnam National University Medical School, Gwang-ju, Republic of Korea; 4 Department of Emergency Medicine, Chonnam National University Medical School, Gwang-ju, Republic of Korea; University of Edinburgh, United Kingdom

## Abstract

**Background:**

Methicillin-resistant *Staphylococcus aureus* (MRSA) is one of the most common causes of infection in the intensive care unit (ICU). Although surveillance culture for MRSA is recommended for ICU patients, no comparative study investigating the optimal sites and frequency of culture has been performed in this population.

**Methods:**

A prospective observational cohort study was performed in an 18-bed emergency intensive care unit (EICU) in a tertiary teaching hospital. A total of 282 patients were included. Samples for MRSA detection were obtained at the time of admission, 48 h after admission, and then weekly thereafter. All subjects were routinely monitored for the development of MRSA infection during their stay in the ICU.

**Results:**

MRSA colonization was detected in 129 (46%) patients over the course of the study. The sensitivity of MRSA surveillance culture was significantly higher in throat or tracheal aspirates (82%; 106/129) than in anterior nares (47%; 61/129) (*P*<0.001). The sensitivity of MRSA surveillance culture for subsequent MRSA infection and MRSA pneumonia was also higher in the throat/trachea (69 and 93%, respectively) than in the anterior nares (48 and 50%, respectively). The area under the curve for subsequent MRSA infection was higher in trachea/throat (0.675) than in the anterior nares (0.648); however, this difference was not significant (*P*>0.05). The area under the curve for MRSA pneumonia was significantly higher in trachea/throat (0.791; 95% CI, 0.739-0.837) than anterior nares (0.649; 95% CI, 0.590-0.705) (*P* = 0.044).

**Conclusion:**

MRSA colonization was more common in the trachea/throat than in the anterior nares in ICU patients. Cultures from throat or tracheal aspirates were more sensitive and predictive of subsequent MRSA pneumonia than cultures from the anterior nares in this population.

## Introduction

Methicillin-resistant *Staphylococcus aureus* (MRSA) is a major human pathogen causing a wide range of both community-onset and nosocomial infections [1]–[3], and the identification of MRSA carriage is therefore an important tool for the control and prevention of these infections. The anterior nares have long been considered the most important site for MRSA colonization, however, recent studies suggest that colonization of the throat is more common than that of the anterior nares among certain populations, suggesting the need for alternative testing protocols [4]–[11].

MRSA is one of the most important pathogens in intensive care units (ICU), causing serious infections resulting in significant morbidity and mortality. For this reason, active surveillance for MRSA is recommended in ICU patients [12], though no consensus has been reached regarding which site to use for optimal detection due to limited data [13]–[15]. Moreover, only a few studies have evaluated the predictive value of surveillance culture for subsequent MRSA infections in ICU patients [16], [17], with no study directly comparing the predictive values based upon surveillance culture site.

The aim of this study was to determine the frequency of MRSA colonization in extra-nasal sites among ICU patients, and to investigate whether traditional surveillance culture of the anterior nares for MRSA shows sufficient sensitivity and predictive value compared to other sites, especially the trachea/throat, in ICU patients.

## Materials and Methods

### Ethics statement

The inclusion criteria and overall study design were approved by the institutional review board (IRB) of Chonnam National University Hospital; due to routine practice for infection control in our hospital, the requirement for written informed consent was waived by IRB.

### Patients

A prospective observational study was performed between March 2010 and February 2011 at the 18-bed emergency intensive care unit (EICU) of Chonnam National University Hospital (Gwang-ju, Republic of Korea), a mixed-ICU consisting of medical (80%) and surgical (20%) patients admitted through the emergency department. All patients >16 years of age admitted to the ICU were included in this study.

### Active surveillance protocol for the detection of MRSA

All patients were screened for the presence of MRSA at the time of admission, 48 h after admission, and then weekly thereafter until MRSA was detected or the patient was discharged from the ICU. Samples were obtained from four sites in each patient: 1) anterior nares; 2) breached skin locations, including catheter insertion sites, pressure sores, and surgical wounds; 3) throat or trachea; and 4) rectum. Swabs of the anterior nares, skin, and rectum were collected using a cotton swab moistened with sterile 0.9% saline by nurses affiliated with either the ICU or office for infection control. A tracheal aspirate was collected using a 22-inch suction catheter in a mucus collector (Specimen trap; Busse, Hauppauge, NY, USA).

Patient samples were inoculated on BBL CHROMagar MRSA medium (BD Diagnostics, Sparks, MD, USA) without broth enrichment for screening [18], [19]. BBL CHROMagar MRSA plates were incubated at 35°C and were reviewed at 24 hrs and again 48 hrs if necessary. Mauve-colored colonies appearing at 24 and 48 hrs were considered MRSA by colony morphology, gram staining, and coagulase testing. The culture was considered negative if there were no mauve-colonies after 48 hrs of incubation. The species and methicillin resistance of all positive isolates were confirmed using either the Vitek 2 (bioMérieux) or Microscan (Dade Behring Inc., Deerfield, IL, USA) automated systems.

### Clinical definitions

Pneumonia was defined as a positive quantitative respiratory culture (>10^4^ colony-forming units [CFU]/mL for bronchoalveolar lavage, and >10^5^ CFU/mL for tracheal aspirate or sputum), and a new or progressive and persistent radiographic infiltrate along with at least two of the following characteristics: fever (>38°C), leukopenia (<4,000 white blood cells/mm^3^) or leukocytosis (>12,000 white blood cells/mm^3^), new onset of purulent sputum or a change in sputum character, and worsening gas exchange [16]. Other ICU-acquired MRSA infections were defined according to the standards set forth by the Modified National Healthcare Safety Network [20].

### Statistical analyses

Categorical variables were compared using Fisher’s exact test, Pearson’s χ^2^ test, or McNemar’s test; continuous variables were compared using either Student’s *t*-test. Variables with *P*-values ≤0.10 in the univariate analysis were included in a multivariate analysis. Multivariate analyses were performed using a logistic regression model in a backward stepwise conditional manner. All tests of significance were two-tailed, with statistical significance defined as *P*-values ≤0.05. All statistical analyses were performed using SPSS (version 19.0; SPSS Inc., Chicago, IL, USA); the area under the receiver operating characteristics (ROC) curves was analyzed using Medcalc (version 11.2; Mariakerke, Belgium).

## Results

### Study population

A total of 295 cases >16 years of age were admitted to the EICU of Chonnam National University Hospital during the study period. Thirteen of the cases represented readmissions and were therefore excluded from the study, resulting in a total of 282 patients analyzed.

### Sensitivity of MRSA surveillance culture from various anatomical sites

MRSA was isolated from one or more anatomical sites in 129/282 (46%) patients over the course of the study. Of these, 59/282 (21%) patients were MRSA-positive at the time of admission; the remaining 70/223 patients (31%) were MRSA-negative at the time of admission, and were therefore colonized during their stay in the ICU. Among the 70 patients who acquired MRSA during their stay in the ICU, 84% (59/70) were culture-positive within the first 48 h following admission and 93% (65/70) culture-positive within the first week. The overall MRSA acquisition rate for all patients included in this study was 21.4 per 1000 patient-days.

**Figure 1:**
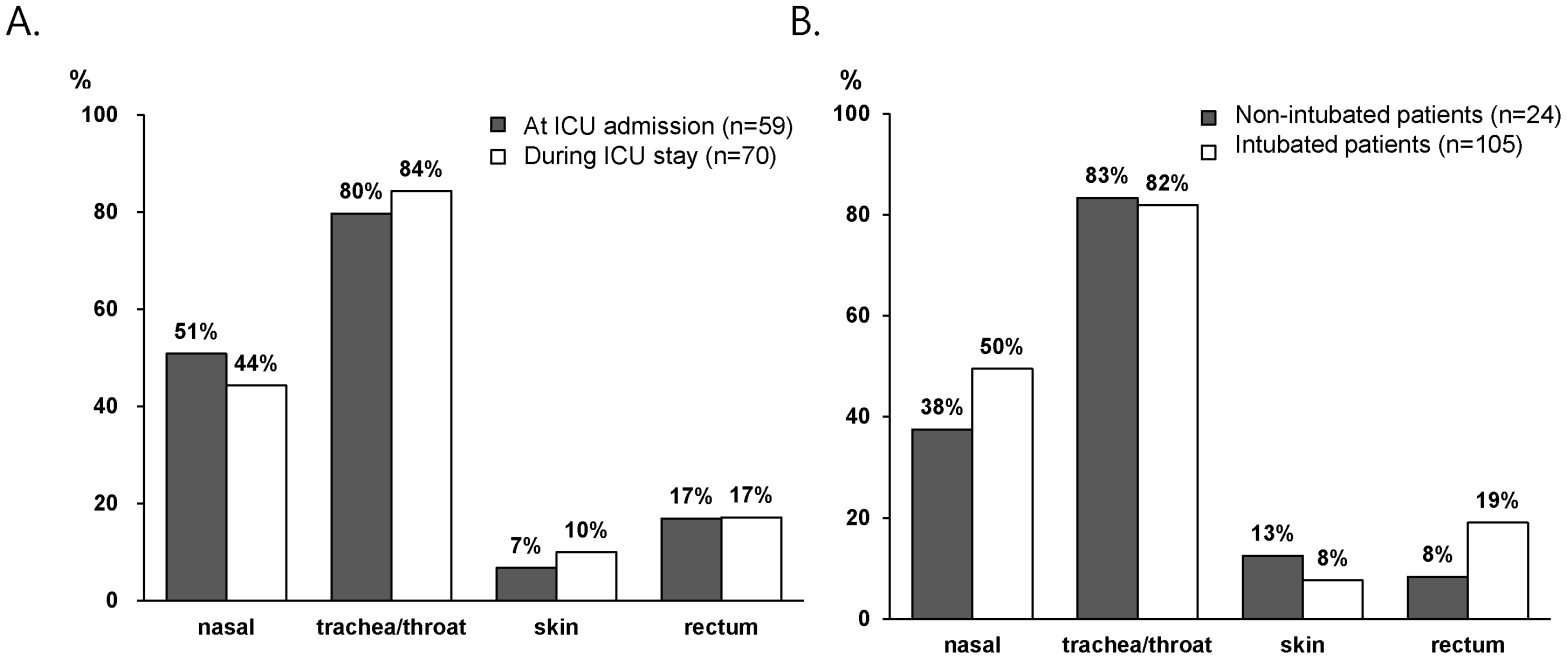
Sensitivity of MRSA surveillance culture for each anatomical site. **A.** Sensitivity of MRSA surveillance culture by collection time. White bars: sensitivity at the time of admission; black bars: sensitivity of cultures collected during the ICU stay. **B.** Sensitivity of MRSA surveillance culture according to intubation. White bars: intubated patients; black bars: non-intubated patients.

The sensitivity of trachea/throat culture (106/129; 82%) was significantly higher than that of anterior nares (61/129; 47%), skin (11/129; 9%), or rectum (22/129; 17%) (Fig. 1; *P*<0.001; McNemar’s test). The sensitivity of MRSA surveillance culture was similar for patients testing positive at the time of admission and those acquiring colonizations during their stay in the ICU for each of the anatomical sites tested (Fig. 1A). The sensitivity was also similar for surveillance cultures performed from throat (20/24; 83%) and tracheal (86/105; 82%) specimens in non-intubated and intubated patients, respectively (Fig. 1B).

### Risk factors associated with MRSA colonization

The risk factors for MRSA colonization in 282 patients included in this study are shown in Table 1. Use of an endotracheal tube and antibiotic use were identified as independent risk factors for MRSA colonization (*P*≤0.05, each).

**Table 1:** Risk factors for MRSA colonization in 282 patients.

Risk factor	Univariate analysis			Multivariate analysis			
	No.(%) of patients		*P* value	*HR*	95% CI		*P* value
	MRSA-negative (N = 153)	MRSA-positive (N = 129)			Lower	Upper	
Antibiotics use within 6-months	29 (19)	85 (66)	<0.001	7.66	4.42	13.27	<0.001
Mechanical ventilation	80 (52)	91 (71)	0.002				
Endotracheal tube	98 (64)	106 (82)	0.001	2.00	1.07	3.72	0.029
Central catheter	101 (66)	102 (79)	0.015				
Nasogastric tube	70 (46)	88 (68)	<0.001				
Cerebral vascular accident	11 (7)	22 (17)	0.010				

**NOTE**. MRSA, methicillin-resistant *Staphylococcus aureus*.

### Value of MRSA surveillance culture from various anatomical sites for predicting MRSA infections

Nosocomial MRSA infections were detected in 10% of patients (29/282) at a rate of 8.0 per 1000 patient-days. For patients with a confirmed MRSA infection, the frequency of MRSA-positive surveillance cultures was significantly higher in patients with vascular catheter-related infections or pneumonia compared with MRSA-negative patients (*P*≤0.05, Table 2).

**Table 2:** MRSA infections detected during the ICU stay.

Characteristics	No.(%) of patients		*P* value
	MRSA-negative (N = 153)	MRSA-positive (N = 129)	
All infections	5 (3)	24 (19)	<0.001
Overall bacteremia	5 (3)	11 (9)	0.057
Vascular catheter-related infection	1 (1)	6 (5)	0.050
Pneumonia	1 (1)	13 (10)	<0.001
Wound or surgical site infection	1 (1)	4 (3)	0.182

**NOTE**. MRSA, methicillin-resistant *Staphylococcus aureus*.

The sensitivity, specificity, and predictive value of MRSA surveillance culture for subsequent MRSA infections are shown for each of the anatomical sites tested (Table 3). The sensitivity and negative predictive value for all MRSA infections and pneumonia were higher for trachea/throat cultures than for anterior nares cultures (Table 3).

**Table 3:** Sensitivity, specificity, and predictive value of MRSA surveillance culture by anatomical site.

Anatomical site	Sensitivity, % (n/N, 95% CI)	Specificity, % (n/N, 95% CI)	Positive Predictive Value, % (n/N, 95% CI)	Negative Predictive Value, % (n/N, 95% CI)
All MRSA infection				
Nasal	48 (14/29, 43–54)	81 (206/253, 77–86)	23 (14/61, 18–28)	93 (206/221, 90–96)
Trachea/throat	69 (20/29, 64–74)	66 (167/253, 61–72)	19 (20/106, 14–24)	95 (167/176, 92–98)
Throat	75 (3/4, 69–81)	77 (57/74, 72–82)	15 (3/20, 11–19)	98 (57/58, 97–100)
Trachea	68 (17/25, 63–73)	62 (110/179, 56–67)	20 (17/86, 15–24)	93 (110/118, 90–96)
All four sites^a^	83 (24/29, 78–87)	59 (148/253, 53–64)	19 (24/129, 14–23)	97 (148/153, 95–99)
MRSA bacteremia				
Nasal	50 (8/16, 44–55)	80 (213/266, 75–85)	13 (8/61, 9–17)	96 (213/221, 94–99)
Trachea/throat	50 (8/16, 44–55)	63 (168/266, 58–69)	8 (8/106, 4–11)	96 (168/176, 93–98)
Throat	50 (1/2, 44–56)	75 (57/76, 70–80)	5 (1/20, 2–8)	98 (57/58, 97–100)
Trachea	50 (7/14, 44–56)	58 (111/190, 53–64)	8 (7/86, 5–11)	94 (111/118, 91–97)
All four sites^a^	69 (11/16, 63–74)	56 (148/266, 50–61)	9 (11/129, 5–12)	97 (148/153, 95–99)
MRSA pneumonia				
Nasal	50 (7/14, 44–56)	80 (214/268, 75–85)	12 (7/61, 8–15)	97 (214/221, 95–99)
Trachea/throat	93 (13/14, 90–96)	65 (175/268, 60–71)	12 (13/106, 9–16)	99 (175/176, 99–100)
Throat	100 (1/1, 100)	75 (58/77, 70–80)	5 (1/20, 2–7)	100 (58/58, 100)
Trachea	92 (12/13, 89–95)	61 (117/191, 56–67)	14 (12/86, 10–18)	99 (117/118, 98–100)
All four sites^a^	93 (13/14, 90–96)	57 (152/268, 51–63)	10 (13/129, 7–14)	99 (152/153, 98–100)

NOTE. MRSA, methicillin-resistant *Staphylococcus aureus*.

a; Nasal + trachea/throat + rectum + skin.

**Figure 2:**
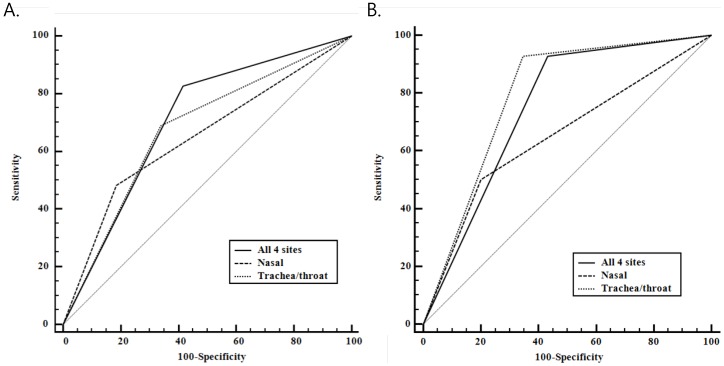
MRSA surveillance cultures as a predictor of MRSA infection by anatomical site. **A.** ROC curve of MRSA surveillance as a predictor of overall MRSA infection. AUC: nasal cultures, 0.648 (95% CI, 0.590-0.704); trachea/throat cultures, 0.675 (95% CI, 0.617-0.729); all 4 sites, 0.706 (95% CI, 0.649-0.759) (*P*>0.05). **B.** ROC curve of MRSA surveillance as a predictor of MRSA pneumonia. AUC: nasal cultures, 0.649 (95% CI, 0.590-0.705); trachea/throat cultures, 0.791 (95% CI, 0.739-0.837); all 4 sites, 0.748 (95% CI, 0.693-0.797).

ROC curves were generated to compare the sensitivity and specificity of MRSA surveillance cultures for each of the anatomical sites tested. The area under the curve (AUC) for subsequent MRSA infection was higher in trachea/throat (0.675) than in the anterior nares (0.648); however, this difference was not significant (Fig. 2A; *P*>0.05). The AUC was significantly higher for trachea/throat cultures (0.791; 95% CI, 0.739-0.837) than for nasal cultures (0.649; 95% CI, 0.590-0.705) for the prediction of MRSA pneumonia (Fig. 2B; *P* = 0.044).

## Discussion

The anterior nares have long been considered the primary niche of *S. aureus* colonization, with surveillance cultures of *S. aureus*, including those for MRSA, performed exclusively from this site. Recent studies [4]–[11], [21] indicate that MRSA colonization may be higher in the throat than in the anterior nares among certain populations; however, these results remain controversial [22], [23]. There have been several studies evaluating the usefulness of throat culture compared to traditional anterior nares culture in ICU patients, and a recent meta-analysis suggested that extra-nasal screening is warranted in ICU patients [21].

In this study, performed in a region with a high prevalence of MRSA infections, we observed far greater sensitivity for surveillance cultures of samples collected from the throat/trachea than in those from the anterior nares. The sensitivity was also greater than that seen in cultures of samples collected from the rectum or skin, two other sites which have been suggested as possible locations of MRSA colonization [21]. Medical devices were found to be a significant risk factor for MRSA colonization. The use of endotracheal and nasogastric tubes was associated with increased MRSA colonization. MRSA, like many other bacterial pathogens, is capable of forming biofilms on medical devices; our data suggest that increased use of medical devices which are placed in the throat and trachea may facilitate MRSA colonization, and could account for the higher prevalence of MRSA observed in these locations.

The data presented here provide valuable information that may directly influence clinical practices in ICUs. Infection control methods for MRSA are primarily based on nasal surveillance, with standard methods of decolonization using mupirocin nasal ointment or chlorhexidine bathing targeting only nasal and skin colonization. However, a recent report suggests that universal decolonization may be more effective than targeted decolonization or screening and isolation at reducing the rate of MRSA infection [24], consistent with the more widespread colonization described here. Our data indicate that the trachea and throat are more common sites of colonization in ICU patients, suggesting that trachea/throat surveillance cultures should be included for better MRSA detection in ICU patients. Additional intervention strategies aimed at decreasing or eradicating tracheal colonization (i.e., material-coated endotracheal tubes) should also be considered.

Several studies have compared the sensitivity of surveillance culture across various anatomical sites in ICU patients and indicated that expanding the frequency of surveillance and the number of anatomical sites tested may increase the predictive value for the detection of multidrug-resistant pathogens [25]–[27]; however, the majority of pathogens detected in these studies were not MRSA, and the heterogeneity in regards to frequency of screening and cost effectiveness makes determining the overall utility of this approach difficult. Few studies evaluated the predictive value of surveillance culture for subsequent MRSA infection [16]–[18] Furthermore, no studies have directly compared the predictive values of surveillance cultures taken from different anatomical sites.

Chan et al. [17] reported that active surveillance culture from multiple sites can accurately exclude MRSA as an etiology in most patients with ventilator-associated pneumonia. This observation is consistent with the results presented here; however, differences in the diagnostic value for each anatomic site were not investigated in that study.

The limitations of nasal MRSA surveillance culture described here are consistent with a prior publication showing nasal colonization with MRSA to be a poor predictor for the subsequent occurrence of MRSA pneumonia or bacteremia [16]. However, trachea/throat MRSA surveillance showed excellent sensitivity and a high negative predictive value for MRSA pneumonia. Our data clearly show that MRSA active surveillance has limited value when performed from the anterior nares only, but is helpful in predicting or excluding subsequent MRSA infections when cultured from extra-nasal sites.

The excellent negative predictive value of trachea/throat surveillance culture can be used to guide empirical therapy against MRSA in patients with suspected ventilator-associated pneumonia beyond the basic search and destroy approach [28], [29]. Trachea/throat surveillance may allow for earlier detection, and therefore earlier initiation of anti-MRSA therapy in pneumonia patients with respiratory MRSA colonization. Moreover, it can reduce the use of unnecessary empirical anti-MRSA therapy in surveillance culture-negative patients, decreasing the likelihood of subsequent colonization with antibiotic-resistant pathogens such as vancomycin-resistant enterococci.

Our study has some limitations. First, we did not perform molecular screening, such as polymerase chain reaction, along with surveillance culture; for this reason, a comparison between molecular screening and culture was not performed. Second, we did not analyze the microbiological characteristics of MRSA isolated from various sites and MRSA detected from a subsequent infection. Third, although lower respiratory tract samples were collected and quantitative culture was performed in all patients suspected for pneumonia, bronchoalveolar lavage was not performed in all patients. Fourth, this study was performed at a single center; further study is needed to confirm and generalize our findings. Fifth, we did not perform throat swabs but rather performed transtracheal aspiration for intubated patients, since nosocomial or ventilator associated pneumonia are common after microaspiration of tracheal contents relating to biofilm formation in the endotracheal tube. In the case of intubated patients, tracheal aspiration can obtain specimens from deeper sites and is more convenient and commonly done than throat culture in ICU.

In conclusion, we found the trachea/throat to be a more common site of MRSA colonization than anterior nares in ICU patients. The sensitivity, negative predictive value, and AUC as a predictor of subsequent MRSA infections were all higher in trachea/throat than in surveillance cultures collected from anterior nares.
